# Generation and characterization of induced pluripotent stem cells of small apes

**DOI:** 10.3389/fcell.2025.1536947

**Published:** 2025-03-19

**Authors:** Yusuke Hamazaki, Hiroto Akuta, Hikaru Suzuki, Hideyuki Tanabe, Kenji Ichiyanagi, Takuya Imamura, Masanori Imamura

**Affiliations:** ^1^ Molecular Biology Section, Center for the Evolutionary Origins of Human Behavior, Kyoto University, Inuyama, Japan; ^2^ Laboratory of Molecular and Cellular Physiology, Graduate School of Integrated Sciences for Life, Hiroshima University, Higashihiroshima, Japan; ^3^ Laboratory of Genome and Epigenome Dynamics, Department of Animal Sciences, Graduate School of Bioagricultural Sciences, Nagoya University, Nagoya, Japan; ^4^ Research Center for Integrative Evolutionary Science, SOKENDAI (The Graduate University for Advanced Studies), Hayama, Japan; ^5^ Department of Medical Neuroscience, Graduate School of Medical Sciences, Kanazawa University, Kanazawa, Japan; ^6^ Sapiens Life Sciences, Evolution and Medicine Research Center, Kanazawa University, Kanazawa, Japan

**Keywords:** small ape, gibbon, siamang, induced pluripotent stem cell, transcriptome

## Abstract

Small apes (family Hylobatidae), encompassing gibbons and siamangs, occupy a pivotal evolutionary position within the hominoid lineage, bridging the gap between great apes and catarrhine monkeys. Although they possess distinctive genomic and phenotypic features—such as rapid chromosomal rearrangements and adaptations for brachiation—functional genomic studies on small apes have been hindered by the limited availability of biological samples and developmental models. Here, we address this gap by successfully reprogramming primary skin fibroblasts from three small ape species: lar gibbons (*Hylobates lar*), Abbott’s gray gibbons (*Hylobates abbotti*), and siamangs (*Symphalangus syndactylus*). Using Sendai virus-based stealth RNA vectors, we generated 31 reprogrammed cell lines, five of which were developed into transgene-free induced pluripotent stem cells. These iPSCs displayed canonical features of primed pluripotency, both morphologically and molecularly, consistent with other primate iPSCs. Directed differentiation experiments confirmed the capacity of the small ape iPSCs to generate cells representing all three germ layers. In particular, their successful differentiation into limb bud mesoderm cells underscores their utility in investigating the molecular and developmental mechanisms unique to small ape forelimb evolution. Transcriptomic profiling of small ape iPSCs revealed significant upregulation of pluripotency-associated genes, alongside elevated expression of transposable elements. Remarkably, *LAVA* retrotransposons—a class of elements specific to small apes—exhibited particularly high expression levels in these cells. Comparative transcriptomic analyses with iPSCs from humans, great apes, and macaques identified evolutionary trends and clade-specific gene expression signatures. These signatures highlighted processes linked to genomic stability and cell death, providing insights into small ape-specific adaptations. This study positions small ape iPSCs as a transformative tool for advancing functional genomics and evolutionary developmental biology. By facilitating detailed investigations into hominoid genome evolution and phenotypic diversification, this system bridges critical gaps in comparative research, enabling deeper exploration of the genetic and cellular underpinnings of small ape-specific traits.

## 1 Introduction

The apes represent the closest extant relatives of humans. As members of the hominoid clade, alongside humans, their genomes contain evolutionary footprints that shed light on the development of human-specific traits. Apes are divided into two distinct groups: the great apes (family Hominidae, which includes bonobos, chimpanzees, gorillas, and orangutans, along with humans) and the small apes (family Hylobatidae, comprising gibbons and siamangs). The small apes represent the earliest lineage to diverge from the common ancestor of hominoids approximately 20–16 million years ago (Mya) (Matsudaira and Ishida, 2010; Thinh et al., 2010; Carbone et al., 2014; Shao et al., 2023). This phylogenetic distinction positions the small apes as a pivotal evolutionary link, bridging the gap between apes and catarrhine monkeys. Following their divergence from the most recent common ancestor, the small apes experienced simultaneous radiation around 5 Mya (Thinh et al., 2010; Carbone et al., 2014), resulting in the diversification of ∼20 species across four genera: *Nomascus*, *Hoolock*, *Hylobates*, and *Symphalangus* (Mootnick and Groves, 2005; Roos, 2016). These species are arboreal and predominantly distributed across the tropical rainforests of East and Southeast Asia (Cunningham and Mootnick, 2009).

Owing to their unique phylogenetic position and evolutionary history, small apes have evolved some characteristics shared with great apes, including morphological traits related to upright body posture and suspensory locomotion, while also evolving distinct physical and social characteristics. These include a smaller body size (5.3–11.9 kg), elongated forelimbs optimized for brachiation, diverse coat color, pair-living, and duet singing (Marshall and Marshall, 1976; Hollihn, 1984; Smith and Jungers, 1997; Mittermeier and Wilson, 2013; Reichard et al., 2016). Their diversification also encompasses the variable chromosome numbers among genera (Müller et al., 2003) accompanied by rapid and large-scale chromosome rearrangements (Carbone et al., 2006). These characteristics make small apes a valuable model for investigating phenotypic diversification and molecular evolution within hominoids. Comparative genomic analyses have been employed to explore the molecular underpinning of these unique traits. Notably, the gibbon genomes harbor the lineage-specific retrotransposons called *LAVA* (*LINE-Alu-VNTR-Alu-like*) (Carbone et al., 2012). The copy number of *LAVA* is estimated to range from 600 to 1,200 within gibbon genomes (Carbone et al., 2012), and these insertions are proposed to contribute to chromosomal rearrangements and genomic plasticity (Carbone et al., 2014; Okhovat et al., 2020). Additionally, genes subject to natural selection (e.g., *TBX5*) (Carbone et al., 2014) and regions that have undergone accelerated evolution (e.g., downstream regions of *DLX5* and *EMX2*) (Carbone et al., 2014; Bi et al., 2023) have been identified as genomic factors potentially underlying the distinct features of small apes. Despite these efforts, linking genomic variations to phenotypic traits remains challenging. Limited access to small ape tissue materials and developmental phenomena (Juan et al., 2023) has hindered functional studies in these species, leaving significant gaps in our understanding of how genomic features translate into unique phenotypic adaptations.

In recent years, induced pluripotent stem cells (iPSCs) have emerged as a groundbreaking experimental platform for functional genomics and evolutionary developmental biology (Juan et al., 2023; Pollen et al., 2023). iPSCs offer several technical advantages, including their derivation from readily accessible tissue cells, such as skin or blood, across a wide range of mammalian species. Moreover, iPSCs exhibit robust proliferation while retaining the capacity to differentiate into derivatives of all three germ layers. These unique properties allow the *in vitro* modeling and manipulation of developmental processes in diverse mammalian species. iPSCs have now been successfully generated for all hominid lineages (humans and great apes). Human iPSCs were established in 2007 (Takahashi et al., 2007), followed by those of chimpanzees and bonobos in 2013 (Marchetto et al., 2013), gorillas in 2014 (Wunderlich et al., 2014), and orangutans in 2015 (Ramaswamy et al., 2015). Great ape iPSCs have been extensively used in cross-species comparisons to investigate molecular and cellular characteristics relative to humans (Dannemann and Romero, 2022). However, despite being members of the hominoid clade, small apes have remained an unexplored lineage in the field of iPSC research (Anwised et al., 2023). Over a decade has passed since the establishment of the first great ape iPSCs, yet small ape iPSCs have not been successfully generated until this year (Bao et al., 2024). Consequently, small apes have represented a phylogenetic gap, or “missing link,” between monkeys and hominoids in the study of human evolutionary developmental biology using iPSCs.

Here, we reprogrammed small ape skin fibroblasts in three species of two genera of Hylobatidae: lar gibbons (*Hylobates lar*), Abbott’s gray gibbons (*Hylobates abbotti*), and siamangs (*Symphalangus syndactylus*). Using Sendai virus (SeV)-based stealth RNA vectors (SRV), we achieved efficient gene transduction, generating a total of 31 reprogrammed cell lines across these three species and subsequently establishing five iPSC lines from lar gibbons and siamangs. The small ape iPSCs were cultured under feeder-free conditions and formed colonies morphologically similar to those of other primate iPSCs. Molecular analysis revealed that these cells exhibited characteristics consistent with the primed pluripotent state. Directed differentiation cultures applied to small ape iPSCs successfully generated neurons, limb bud mesoderm cells, and definitive endoderm cells, demonstrating their differentiation potential. Furthermore, cross-species comparative analyses of gene expression between small ape and other primate iPSCs highlighted clade-specific and evolutionary trends within primates. These small ape iPSCs, along with their directed differentiation cultures, offer a valuable platform for elucidating the evolutionary and developmental biology of hominoids, providing critical insights into their unique biological and evolutionary adaptations.

## 2 Materials and methods

### 2.1 Primary culture of small ape skin fibroblasts

Skin specimens were obtained from two lar gibbons (*H. lar*) [GAIN-ID: 0023 (Jessica, a 24-year-old female) and GAIN-ID: 0247 (Kyutaro, a 21-year-old male)], one Abbott’s gray gibbon (*H. abbotti*) [GAIN-ID: 0159 (Anna, an estimated 46-year-old female)], and two siamangs (*S. syndactylus*) [GAIN-ID: 0105 (Matsu, an estimated 32-year-old female) and GAIN-ID: 0297 (Peach, an 11-year-old female)]. These samples were provided by the Japan Monkey Centre, Toyohashi Zoo and Botanical Park, and the Great Ape Information Network (GAIN) for primary cultures. Each skin specimen was minced into small pieces and transferred onto tissue culture dishes (353,002; Corning, Glendale, AZ, United States) with 15% fetal bovine serum (FBS)/Dulbecco’s modified Eagle’s medium (DMEM), which consists of DMEM with high glucose (044-29765; Wako, Osaka, Japan) supplemented with 15% FBS, 1× non-essential amino acids (139-15651; Wako), 1× GlutaMAX Supplement (35050061; Gibco, Waltham, MA, United States), 1 mM sodium pyruvate (190-14881; Wako), 0.11 mM 2-mercaptoethanol (21,985-023; Gibco), and 100 units/mL penicillin and 100 μg/mL streptomycin (168-23191; Wako). Fibroblast outgrowth from the skin pieces was observed after approximately 1 week of culture. The fibroblasts were subsequently passaged though single-cell dissociation with 0.25% trypsin/ethylenediaminetetraacetic acid (EDTA) and expanded in 15% FBS/DMEM medium on 0.1% gelatin-coated culture dishes at 37°C with 5% CO_2_.

### 2.2 Reprogramming of small ape fibroblasts with SRVs

Small ape skin fibroblasts (passage number 2–4) were seeded at 1.0 × 10^5^ cells/well on 0.1% gelatin-coated culture plates. The following day, the cells were transduced with six human reprogramming factors (OCT3/4, KLF4, SOX2, c-MYC, LIN28, and NANOG) using SRV iPSC-3 (S1011626A; Tokiwa Bio, Tsukuba, Japan) at a multiplicity of infection (MOI) of three for GAIN-ID: 0247, 0159, 0105, and 0297. For GAIN-ID: 0023, transduction was performed with four human reprogramming factors (OCT3/4, KLF4, SOX2, and c-MYC) using the SRV iPSC-1 (S1011624A; Tokiwa Bio) at an MOI of 3. The transduction procedure involved incubating the cells with the respective SRVs at room temperature for 2 h, followed by overnight incubation at 37°C with 5% CO_2_. Twenty-four hours after transduction, the cells were dissociated with 0.25% trypsin/EDTA and reseeded onto iMatrix-511-coated 24-well culture plates at 1.0 × 10^4^ cells/well. The medium was changed every other day under two different conditions. For condition A, cells were cultured in 15% FBS/DMEM medium from day 1 to day 7, followed by StemFit AK02N medium (AK02N; Ajinomoto, Tokyo, Japan) without supplements from day 7 to approximately day 21. Thereafter, the cells were maintained in StemFit AK02N medium. For condition B, StemFit AK02N medium without supplements was used from day 1 to day 7 or 14, followed by continued culture in StemFit AK02N medium. Emerging EGFP^+^ reprogrammed colonies were manually picked and cultured in StemFit AK02N medium on iMatrix-511-coated culture plates.

### 2.3 Removal of SRVs by siRNA transduction

To remove SRVs from expanded EGFP^+^ reprogrammed cells, small interfering RNA (siRNA) transfection was performed in accordance with the manufacturer’s instructions (S1011626A; Tokiwa Bio). Briefly, EGFP^+^ reprogrammed cells, after several serial passages, were dissociated with TrypLE Express (12604021; Gibco) and seeded at 0.75 × 10^4^ cells/well in StemFit AK02N medium supplemented with 10 μM Y-27632 (036-24023; Wako) on iMatrix-511-coated 24-well culture plates. The next day, the medium was replaced with fresh StemFit AK02N medium. The cells were transfected with siRNA (sense: CAAUAGUUCACGCUGAAAGUG; anti-sense: CUUUCAGCGUGAACUAUUGCU) using Lipofectamine RNAiMAX (13778100; Thermo Fisher Scientific, Waltham, MA, United States) on days 1 and 4 after plating. The siRNA-transfected cells were passaged upon reaching sub-confluency and maintained in StemFit AK02N medium on iMatrix-511-coated culture plates. For subcloning SRV-free iPSCs, the siRNA-transfected cells were seeded at 0.5 × 10^3^ cells/cm^2^, and EGFP^−^ colonies were manually picked up and expanded as iPSCs in StemFit AK02N medium on iMatrix-511-coated culture dishes.

### 2.4 Small ape iPSC maintenance

Small ape iPSCs were maintained in StemFit AK02N medium on iMatrix-511-coated culture dishes. The medium was refreshed with StemFit AK02N medium on days 1, 3, 4, and 5 following passage. On day 6, the iPSCs were dissociated with TrypLE Express and reseeded at 1.4 × 10^3^ cells/cm^2^ onto iMatrix-511-coated culture plates in StemFit AK02N medium supplemented with 10 μM Y-27632.

### 2.5 Chromosome analysis

Metaphase spreads of each small ape iPSC line were prepared following the standard procedure (Tanabe et al., 2000). Briefly, KaryoMAX Colcemid Solution (15210-040; Gibco) was added to iPSC cultures at a final concentration of 0.05 μg/mL for 1.5–3 h. The harvested cells were treated with a hypotonic solution of 0.075 mol/L KCI containing 0.1% (w/v) trisodium citrate for 22 min at 37°C, followed by fixation with Carnoy’s fixative. The cell suspensions were then dispensed onto glass slides using a HANABI metaphase spreader (ADSTEC), air-dried, and stored in a freezer until further analyses. Chromosome numbers and structural rearrangements were evaluated using the QFH-banding technique (Yoshida et al., 1975), which involves staining with Quinacrine mustard and Hoechst 33258. Over 20 QFH-banded metaphase images were captured and analyzed for each iPSC line using a Leica DM5000B fluorescence microscope equipped with a CCD camera and CW4000 image analysis system (Leica Microsystems). Chromosomes of the lar gibbon (*H. lar*) and siamang (*S. syndactylus*) were identified and assigned based on the *Atlas of Mammalian Chromosomes* (Graphodatsky et al., 2020) and related references (Jauch et al., 1992; Capozzi et al., 2012; Stanyon, 2013).

### 2.6 RT-PCR

Total RNA was extracted with RNeasy Plus Mini Kit (74,104; Qiagen, Hilden, Germany) and reverse-transcribed into cDNA with the PrimeScript FAST RT Reagent Kit with gDNA Eraser (RR092A; TaKaRa, Kusatsu, Japan). RT-PCR analyses were performed semi-quantitatively with ExTaq Hot Start Version (RR006A; TaKaRa). Quantitative RT-PCR analyses were performed with THUNDERBIRD Next SYBR qPCR Mix (QPS-201; Toyobo, Osaka, Japan) on a StepOne Real-Time PCR System (Applied Biosystems, Foster City, CA, United States). Primer sequences are provided in Supplementary Table S1.

### 2.7 RNA sequencing analyses

Total RNA was extracted using ISOGEN (317-02503; Nippon Gene, Tokyo, Japan) and Direct-Zol RNA Miniprep (R2050; Zymo Research, Irvine, CA, United States). cDNA library preparation and sequencing were performed by Azenta Japan (Tokyo, Japan). Briefly, cDNA libraries were constructed using NEBNext Ultra Directional RNA Library Prep Kit for Illumina (E7420; New England Biolabs, Ipswich, MA, United States) and sequenced on an Illumina NovaSeq with a 2 × 150 bp paired-end configuration. Each sample yielded approximately 20 million sequencing reads.

For gene expression profiling of small ape iPSCs, Trim Galore was used for adapter and quality trimming with the -fastqc option (https://zenodo.org/records/7598955). Sequencing reads from small ape cells were mapped to nomLeu3 using STAR (Dobin et al., 2013), and uniquely mapped reads were retained for further analyses. BAM files were converted to BED format, and PCR duplicates were removed. The intersectBed tool in Bedtools was used to sum reads of overlapping exons of genes homologous to those of hg38 (Quinlan and Hall, 2010). Only the first read of paired-end reads was included in the count. Transcripts per million (TPM) values were calculated for normalization using Pandas (McKinney, 2010). Principal component analysis (PCA) and hierarchical clustering were conducted using Python 3.10.13.

For transposable element (TE) expression profiling, the consensus sequence of *LAVA* was obtained from a previous study (Carbone et al., 2012), and input into repeatmasker with the default parameters. Full-length (1,300 bp) and nearly full-length copies were selected on the basis of the following criteria: repStart <100 and repEnd >1,200. These copies were added to the RepeatMasker track of nomLeu3, while repeat annotations overlapping with *LAVA* were discarded. TE expression analysis was performed in accordance with our previous study (Hirata et al., 2022). Briefly, sequencing reads were mapped to nomLeu3 using Hisat2 with the--no-spliced-alignment option. For genomic regions with multiple hits, one candidate region was randomly selected. Only reads aligned in the sense orientation with the RepeatMasker track were used and normalized as reads per million reads mapped to the genome (RPM) to evaluate the expression levels.

For cross-species analyses, previously published fastq files of primate iPSC transcriptomes (PRJNA206563, PRJEB65856, PRJNA329031, PRJNA218873, PRJNA649770, PRJNA830828, PRJNA987625) were used (Marchetto et al., 2013; Wunderlich et al., 2014; Thoma et al., 2016; Hirata et al., 2022; Roodgar et al., 2022; Li et al., 2024; Ruiz-Orera et al., 2024). Preprocessed reads were mapped to the reference genomes of each species (hg38, panTro6, panPan3, gorGor6, ponAbe3, nomLeu3, rheMac10, macFas5) using STAR. Annotations from hg38 were transferred to other reference genomes using LiftOver (Hinrichs et al., 2006) to generate reference annotations based on hg38 (Hinrichs et al., 2006). Transcripts transferred across all seven non-human primate reference genomes (131,175 transcripts) and protein-coding genes commonly annotated across all eight reference genomes (13,543 genes) were used. For samples derived from the same individuals, reads mapped to the same genes were combined. Genes differentially expressed between each BioProject were identified using EdgeR [false detection rate (FDR) < 0.05] and excluded from further analysis to reduce batch effects (Robinson et al., 2010). This filtering resulted in a dataset containing 5,310 genes for cross-species analyses. Hierarchical clustering was performed using TPM value and the hclust function in the stats R package. FDR was calculated using EdgeR, and genes with FDR <0.05 and |log_2_FC| > 1 were classified as being differentially expressed. Gene ontology analysis was performed using DAVID (Huang et al., 2009; Sherman et al., 2022).

### 2.8 Alkaline phosphatase staining and immunofluorescence analyses

Alkaline phosphatase staining was performed using Leukocyte Alkaline Phosphatase kit (86R-1KT; Sigma-Aldrich, St. Louis, MO, United States), in accordance with the manufacturer’s instructions. For immunofluorescence staining, cells (passage number over 9) were fixed with 4% paraformaldehyde (PFA), permeabilized with 0.5% Triton X-100, and blocked with 20% ImmunoBlock (CTKN001; KAC, Kyoto, Japan). The cells were then incubated overnight with primary antibodies: OCT3/4 (611,202; BD Biosciences; 1:150), SOX2 (MAB 2018; R&D Systems, Minneapolis, MN, United States; 1:150), NANOG (ab109250; Abcam, Cambridge, United Kingdom; 1:150), SSEA4 (MAB4304; Millipore, Burlington, MA, United States; 1:150), R-10G (011-25811; Wako; 1:150), tubulin β3 (801201; BioLegend, San Diego, CA, United States; 1:150), HAND1 (AF3168-SP; R&D Systems; 10 μg/mL), PRRX1 (ZRB2165; Sigma-Aldrich; 1:200), and goat anti-SOX17 (AF 1924; R&D Systems; 1:150). After washing with PBS, the cells were incubated with secondary antibodies: Alexa Fluor 488 goat anti-mouse IgG (A32723; Thermo Fisher Scientific; 1:500), Alexa Fluor 488 donkey anti-goat IgG (A11055; Thermo Fisher Scientific; 1:500), and Alexa Fluor 555 donkey anti-rabbit IgG (A31572; Thermo Fisher Scientific; 1:500). Nuclei were counterstained with 1 μg/mL 4′,6-diamidino-2-phenylindole (DAPI) (D523; Dojindo, Kumamoto, Japan). Each experiment included negative controls without the primary antibodies. Images were acquired using a BZ-X700 (Keyence, Osaka, Japan).

### 2.9 Direct neurosphere formation and neuronal differentiation

Direct neurosphere formation culture was performed following modified versions of previously reported procedures (Nakai et al., 2018; Kitajima et al., 2020; Nakai et al., 2022). Small ape iPSCs were dissociated with TrypLE Express and plated at 9.0 × 10^3^ cells/well into 96-well low-attachment culture plates (174,929; Thermo Fisher Scientific) in KBM Neural Stem Cell medium (16050200; Kohjin Bio, Sakado, Japan) supplemented with 10 μM Y-27632. The next day, the medium was further supplemented with 2 μM dorsomorphin (11967; Cayman Chemical Company, Ann Arbor, MI, United States), 10 μM SB431542 (13,031; Cayman Chemical Company), 1 ng/mL Stembeads FGF2 (SB500; StemCultures, Rensselaer, NY, United States), and 1× B-27 supplement (17504044; Gibco). On day 4, half of the medium was replaced with fresh medium. For neuronal differentiation, day 7 neurospheres were attached onto Geltrex (A1569601; Gibco)-coated culture plates and cultured for 2 weeks in KBM Neural Stem Cell medium without fibroblast growth factor (FGF)2 and epidermal growth factor (EGF), but supplemented with 1× B-27 supplement.

### 2.10 Limb bud mesoderm differentiation

Limb bud mesoderm differentiation was performed with reference to previous reports (Loh et al., 2016; Yamada et al., 2021; Smith et al., 2023). Small ape iPSCs were dissociated with TrypLE Express and seeded at 1.5 × 10^3^ cells/cm^2^ onto iMatrix-511-coated culture plates in StemFit AK02N medium supplemented with 10 μM Y-27632. The medium was replaced with fresh StemFit AK02N medium the next day. After another 2 days, the iPSCs were washed with DMEM and cultured in chemically defined CDM2 basal medium supplemented with 30 ng/mL Activin A (014-27621; Wako), 6 μM CHIR99021 (13,122; Cayman Chemical Company), 40 ng/mL human BMP4 (120-05ET; Peprotech, Cranbury, NJ, United States), 20 ng/mL human FGF2 (13,256-029; Gibco), and 100 nM PIK90 (S1187; Selleck, Houston, TX, United States) for 24 h to induce mid primitive streak differentiation (days 0–1). The next day, the mid primitive streak cells were washed with DMEM and cultured in CDM2 basal medium with 1 μM A83-01 (S7692; Selleck), 1 μM Wnt-C59 (S7037; Selleck), and 30 ng/mL human BMP4 for 24 h to induce lateral plate mesoderm differentiation (days 1–2). Then, the lateral plate mesoderm cells were washed with DMEM and cultured in CDM2 basal medium with 3 μM CHIR99021, 30 ng/mL human BMP2 (120-02; Peprotech), 10 ng/mL human FGF2, and 1× B-27 supplement for 48 h to induce limb bud mesoderm differentiation (days 2–4). The limb bud mesoderm differentiation medium was replaced daily with fresh medium. The CDM2 basal medium was composed of a 1:1 mixture of Iscove’s Modified Dulbecco’s Medium (IMDM, 31980030; Gibco) and Hams’ F-12 (31765035; Gibco) supplemented with 1 mg/mL polyvinyl alcohol (P8136; Sigma-Aldrich), 1% CD lipid concentrate (11905031; Gibco), 450 nM monothioglycerol (195-15791; Wako), 7 μg/mL human insulin (093-06351; Wako), 15 μg/mL transferrin (201-18081; Wako), and 100 units/mL penicillin and 100 μg/mL streptomycin.

### 2.11 Definitive endoderm differentiation

Definitive endoderm differentiation was performed with reference to previous reports (Loh et al., 2014; Ang et al., 2018). Small ape iPSCs were dissociated with TrypLE Express and seeded at 1.5 × 10^4^ cells/cm^2^ onto iMatrix-511/111 (1:3 mixture of iMatrix-511 and iMatrix-111)-coated culture plates in StemFit AK02N medium supplemented with 10 μM Y-27632. The medium was replaced with a fresh StemFit AK02N medium the next day. One day later, the iPSCs were washed with DMEM and cultured in endoderm induction medium supplemented with 100 ng/mL Activin A, 3 μM CHIR99021, 10 ng/mL human FGF2, and 100 nM PIK90 for 24 h to induce anterior primitive streak differentiation (days 0–1). On day 1, anterior primitive streak cells were washed with DMEM and cultured in endoderm induction medium supplemented with 100 ng/mL Activin A, 500 nM LDN193189 (S2618; Selleck), and 100 nM PIK90 for 24 h to induce definitive endoderm differentiation (days 1–2). The endoderm induction medium was composed of DMEM/F-12 (048-29785; Wako) supplemented with 0.5× B-27 supplement minus vitamin A (12587010; Gibco), 0.5× N-2 supplement (141-08941; Wako), 1×GlutaMAX supplement, and 100 units/mL penicillin and 100 μg/mL streptomycin.

## 3 Results

### 3.1 Generation of small ape iPSCs by SRV-based transduction of human reprogramming factors

**FIGURE 1:**
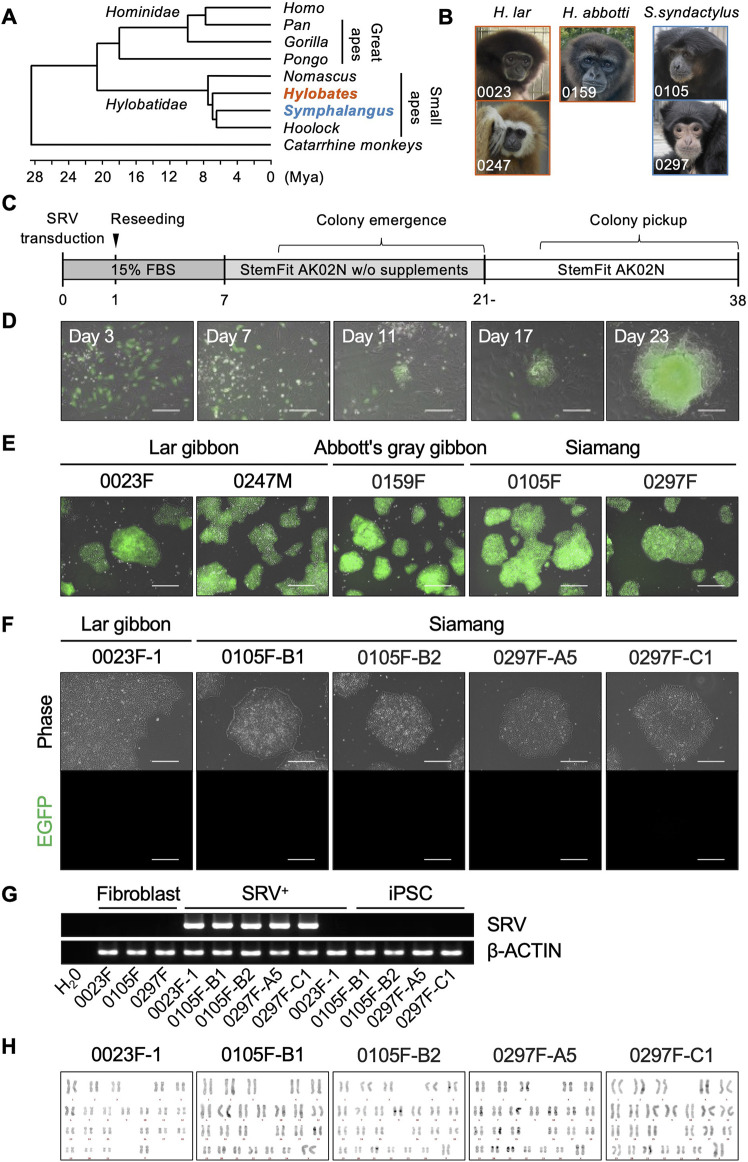
Generation of small ape iPSCs. **(A)** Phylogenetic relationships within the hominoid clade, referring to a previous report (Shao et al., 2023). **(B)** Photographs of donor individuals whose fibroblasts were used for reprogramming. Primary fibroblasts were cultured from skin specimens collected from two lar gibbons [*Hylobates lar* (*H. lar*), individual ID: 0023 and 0247], one Abbott’s gray gibbon [*Hylobates abbotti* (*H. abbotti*), individual ID: 0159], and two siamangs [*Symphalangus syndactylus* (*S. syndactylus*), individual ID: 0105 and 0297]. **(C)** Schematic overview of the process of reprogramming small ape fibroblasts. **(D)** Merged phase-contrast and EGFP fluorescence images of SRV-transduced cells and emerging colonies during reprogramming. Scale bar: 300 μm. **(E)** Merged phase-contrast and EGFP fluorescence images of representative SRV^+^ reprogrammed cell lines after colony picking and expansion. Scale bar: 300 μm. **(F)** Phase-contrast and EGFP fluorescence images of small iPSC lines established after SRV removal. Scale bar: 300 μm. **(G)** RT-PCR analysis of SRVs in parental fibroblasts (Fibroblast), SRV^+^ reprogrammed cells (SRV^+^), and iPSCs. *β-ACTIN* was used as an internal control. **(H)** QFH-banding analysis of chromosomes obtained from metaphase spreads of small ape iPSCs. Chromosomal analyses were conducted on more than 10 metaphase spreads for each iPSC line. The normal karyotype is 2n = 44 for lar gibbons and 2n = 50 for siamangs.

Small apes (family Hylobatidae) occupy distinct phylogenetic branches positioned between hominoids (humans and great apes) and catarrhine monkeys. Their rapid diversification has led to their classification into four genera: *Nomascus*, *Hoolock*, *Hylobates*, and *Symphalangus* (Mootnick and Groves, 2005; Carbone et al., 2014; Roos, 2016; Shao et al., 2023) (Figure 1A). For the induction of iPSCs, primary fibroblast cells were cultured from skin specimens collected from five individuals representing three species within two genera: two lar gibbons (*H. lar*, 0023F and 0247M) and one Abbott’s gray gibbon (*H. abbotti*, 0159F) in the genus *Hylobates*, and two siamangs (*S. syndactylus*, 0105F and 0297F) in the genus *Symphalangus* (Figure 1B; Supplementary Figure S1).

The SRV, derived from SeV, was employed to deliver reprogramming factors into the small ape fibroblasts. SeV targets sialic acids on cellular membranes, facilitating efficient integration across diverse mammalian species (Bitzer et al., 2003). SRV offers the additional advantages of high and stable transgene expression because it replicates within the cytoplasm of transduced cells unless actively eliminated by small interfering RNA (siRNA). Using SeV vectors and SRVs, we have established iPSCs in non-human primates such as chimpanzees and Japanese macaques (Nakai et al., 2018; Imamura et al., 2024). For this study, SRVs encoding either four (*OCT4*, *KLF4*, *SOX2*, and *c-MYC*) or six (*OCT4*, *KLF4*, *SOX2*, *c-MYC*, *NANOG*, and *LIN28*) human reprogramming factors, along with an *enhanced green fluorescent protein* (EGFP) reporter gene (Nakanishi and Iijima, 2024), were used to reprogram small ape fibroblasts (Figure 1C). Three days after SRV transduction, the successful delivery of transgenes was confirmed *via* EGFP fluorescence, which indicated efficient gene transduction into small ape fibroblasts. Although most EGFP^+^ cells underwent cell death over the course of subsequent culture, a small number of tightly packed cell colonies, exhibiting EGFP fluorescence, emerged between days 10 and 21 (Figure 1D). These EGFP^+^ colonies continued to proliferate and were manually picked up around day 30. Eventually, 1–14 proliferating EGFP^+^ cell lines (designated as SRV^+^ reprogrammed cells) were independently established from each of the five small ape individuals (Table 1).

**TABLE 1:** Information on donors and the cell lines established in this study.

Species	GAIN-ID	Sex	Age	SRV^+^ (Condition A, Condition B)	iPSC (Condition A, Condition B)
Lar gibbon (*Hylobates lar*)	0023	Female	24	(1, NT)	(1, NT)
Lar gibbon *(Hylobates lar*)	0247	Male	21	(8, NT)	(0, NT)
Abbott’s gray gibbon (*Hylobates abbotti*)	0159	Female	Estimated 46	(0, 4)	(NT, ND)
Siamang (*Symphalangus syndactylus*)	0105	Female	Estimated 32	(4, NT)	(2, NT)
Siamang (*Symphalangus syndactylus*)	0297	Female	11	(2, 12)	(1, 1)

NT (not tested), no experiments were conducted; ND (not determined), no iPSCs, were established using one SRV^+^ reprogrammed cell line, while the remaining three cell lines have not yet been tested.

The SRV^+^ reprogrammed cells derived from small ape fibroblasts could be expanded in StemFit AK02N medium under feeder-free conditions with the laminin 511 E8 fragment (iMatrix-511) coating (Nakagawa et al., 2014) (Figure 1E). After several passages, selected SRV^+^ reprogrammed cell lines underwent siRNA transfection to eliminate the transduced SRVs, resulting in the emergence of EGFP^−^ colonies (data not shown). Subsequent cloning of these EGFP^−^ colonies led to the establishment of five small ape iPSC lines: one line (0023F-1) from a lar gibbon and four lines (0105F-B1, 0105F-B2, 0297F-A5, and 0297F-C1) from two siamangs. These small ape iPSCs were maintained in StemFit AK02N medium on iMatrix-511, under conditions identical to those used for human and other non-human primate iPSCs (Nakagawa et al., 2014; Kitajima et al., 2020; Imamura et al., 2024), and exhibited no detectable EGFP fluorescence (Figure 1F). The absence of SRVs was further validated by RT-PCR (Figure 1G). Chromosome QFH-banding confirmed normal karyotypes for the iPSC lines, with lar gibbons exhibiting 2n = 44 and siamangs 2n = 50 (Figure 1H).

### 3.2 Pluripotency gene expression characteristics of the small ape iPSCs

**FIGURE 2:**
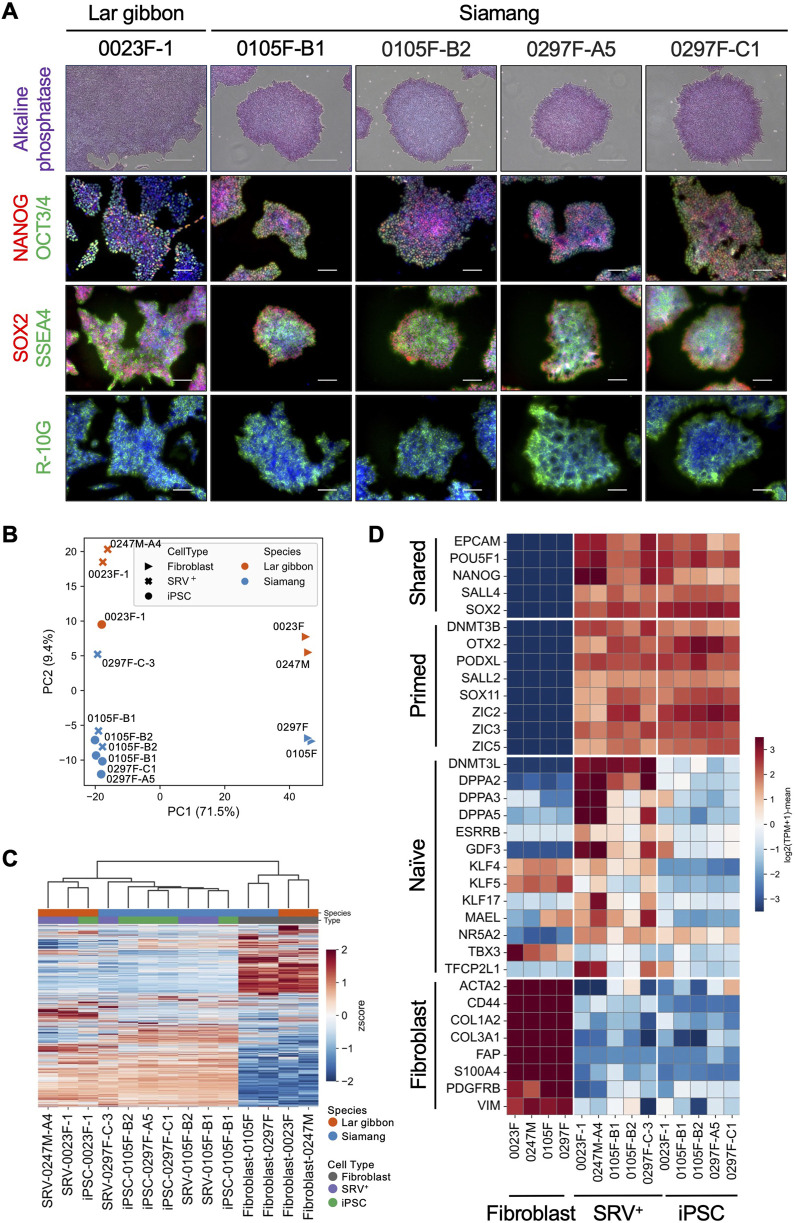
Expression of pluripotency genes in small ape iPSCs. **(A)** Alkaline phosphatase (AP) staining and immunofluorescence images of pluripotency markers (OCT3/4, NANOG, SOX2, SSEA4, R-10G) in lar gibbon (0023F-1) and siamang (0105F-B1, 0105F-B2, 0297F-A5, 0297F-C1) iPSCs. Nuclei were counterstained with DAPI. Scale bar: 300 μm (AP staining) or 100 μm (immunofluorescence). **(B–D)** RNA-seq analyses of small ape fibroblasts, SRV^+^ reprogrammed cells, and iPSCs. **(B)** PCA of the transcriptomes. **(C)** Heatmap and hierarchical clustering of transcriptomes. **(D)** Heatmap of the expression of shared, primed, and naïve pluripotency genes, as well as fibroblast-related genes.

To evaluate the pluripotency characteristics of the established small ape iPSCs, we analyzed the expression of key pluripotency-associated genes in iPSCs derived from a lar gibbon and siamangs. All of the small ape iPSC lines demonstrated alkaline phosphatase activity and were immunopositive for core pluripotency-associated transcription factors (OCT3/4, NANOG, and SOX2), as well as cell surface antigens (SSEA4 and R-10G) (Figure 2A; Supplementary Figure S2). To further characterize their transcriptional profiles, we conducted RNA-seq analyses on parental fibroblasts, SRV^+^ reprogrammed cells, and iPSCs. Principal component analysis (PCA) revealed a clear separation between cells before reprogramming (fibroblasts) and after reprogramming (SRV^+^ reprogrammed cells and iPSCs) along the first principal component (PC1) (Figure 2B). The top 100 genes contributing to PC1 included core pluripotency genes (e.g., *LIN28A*, *SOX2*, *POU5F1*, *PODXL*, *ZIC2*, *OTX2*, *NANOG*, *MYCN*, and *SALL4*) as well as fibroblast-related genes (e.g., *ASPN*, *COL3A1*, *COL6A3*, *POSTN*, *CDH13*, *DCN*, *LOX*, S100A4, and *COL8A1*) (Supplementary Table S2). These results confirmed the successful reprogramming of small ape fibroblasts through SRV-based gene transduction. The separation along the second principal component (PC2) appeared to reflect interspecies differences between lar gibbons and siamangs (Figure 2B). This distinction was further supported by unsupervised hierarchical clustering, which revealed a similar grouping pattern (Figure 2C).

We further examined the pluripotency state of the small ape iPSCs by analyzing the expression of genes associated with naïve or primed pluripotency using RNA-seq profiles (Takashima et al., 2014; Endoh and Niwa, 2022; Kunitomi et al., 2022). Consistent with the PC1 components identified in the PCA, genes associated with “shared pluripotency”—those common to both naïve and primed states (e.g., *EPCAM*, *POU5F1*, and *NANOG*)—were expressed in SRV^+^ reprogrammed cells and iPSCs, but were absent from fibroblasts (Figure 2D). Conversely, fibroblast-related genes (e.g., *ACTA2*, *CD44*, and *COL1A2*) were expressed in fibroblasts but not in SRV^+^ reprogrammed cells or iPSCs. Primed pluripotency genes (e.g., *DNMT3B*, *OTX2*, and *PODXL*) exhibited an expression pattern similar to that of the shared pluripotency genes, further indicating the transition to a primed pluripotent state. In contrast, naïve pluripotency genes (e.g., *DNMT3L*, *DPPA2*, and *DPPA3*) showed minimal expression in the iPSCs, reinforcing their classification as primed pluripotent cells. Interestingly, partial expression of naïve pluripotency genes was observed in the SRV^+^ reprogrammed cells, potentially attributable to the constitutive high expression of SRV-derived reprogramming factors. This observation aligns with findings in other species, where forced expression of reprogramming factors induces naïve-like pluripotency signatures (Fang et al., 2015; Honda et al., 2017a; Honda et al., 2017b; Kisa et al., 2017; Shiozawa et al., 2020).

### 3.3 Transposon expression of the small ape iPSCs

**FIGURE 3:**
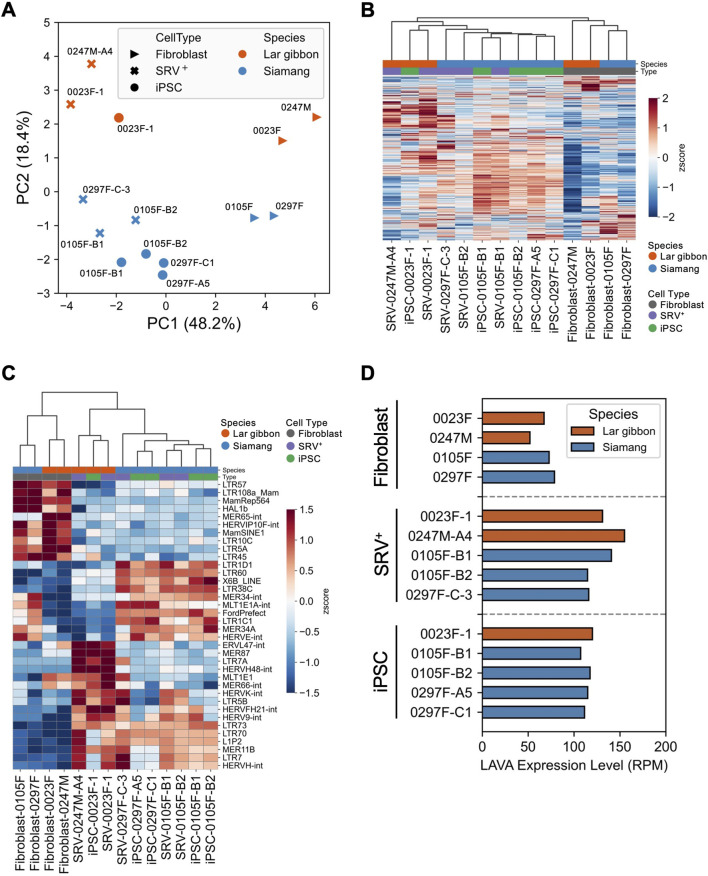
Expression of transposable elements in small ape iPSCs. **(A–D)** RNA-seq analyses of the expression of transposable elements (TEs) in small ape fibroblasts, SRV^+^ reprogrammed cells, and iPSCs. **(A)** PCA of TE expression. **(B)** Heatmap and hierarchical clustering of TE expression. **(C)** Heatmap and hierarchical clustering of the expression of the top 36 PC-loading TEs. **(D)** Expression analysis of the small ape-specific retrotransposon *LAVA*.

To further dissect the molecular characteristics of the small ape iPSCs, we conducted RNA-seq analysis with a specific focus on transposable elements (TEs) (Supplementary Table S3). PCA of overall TE expression revealed distinct separation along PC1 based on reprogramming status and along PC2 based on species differences (Figure 3A). Notably, overall TE expression was upregulated following reprogramming (Figure 3B). This upregulation aligns with findings in early development, reprogramming, and pluripotent stem cells, where TE expression is typically elevated, contrasting with its suppression in somatic cells (Feng et al., 2010; Göke et al., 2015; Grow et al., 2015). The observed increase in TE expression further supports the assertion that the small ape fibroblasts were successfully reprogrammed, as corroborated by the upregulation of pluripotency-associated genes (Supplementary Table S2; Figure 2D). To identify TEs differentially expressed across reprogramming states and species, we extracted the top 10 TEs with the highest positive and negative loadings from PC1 and PC2. This analysis identified 36 TEs, with four overlapping between the components (*HERV-K-int*: PC1-negative/PC2-positive, *MER65-int*: PC1-positive/PC2-positive, *HERVF-H21-int*: PC1-negative/PC2-positive, *HERV-H48-int*: PC1-negative/PC2-positive) (Figure 3C). Among these, several endogenous retrovirus (ERV) elements exhibited higher expression in SRV^+^ reprogrammed cells and iPSCs across both species. Notable examples included *HERV-H* and *LTR7*, which are also highly expressed in human pluripotent stem cells (Santoni et al., 2012; Ohnuki et al., 2014), indicating evolutionary conservation of these expressional regulatory mechanisms among primates. Conversely, certain ERV and ancient repeat elements, such as *LTR5A* and *Mamrep564*, displayed higher expression in fibroblasts than in SRV^+^ reprogrammed cells and iPSCs. Additionally, species-specific expression patterns were identified for some ERV elements, with consistent expression levels before and after reprogramming (e.g., *MLT1E1*, *MER66-int*, *MER34-int*, *HERVE-int*). Finally, we examined the expression of *LAVA*, a TE unique to small apes and absent from other primate genomes (Carbone et al., 2012). *LAVA* was upregulated in SRV^+^ reprogrammed cells and iPSCs compared with the level in fibroblasts (Figure 3D), consistent with the behavior of other TEs conserved among primate genomes (Figure 3B).

### 3.4 Directed differentiation of the small ape iPSCs into ectoderm, mesoderm, and endoderm lineages

**FIGURE 4:**
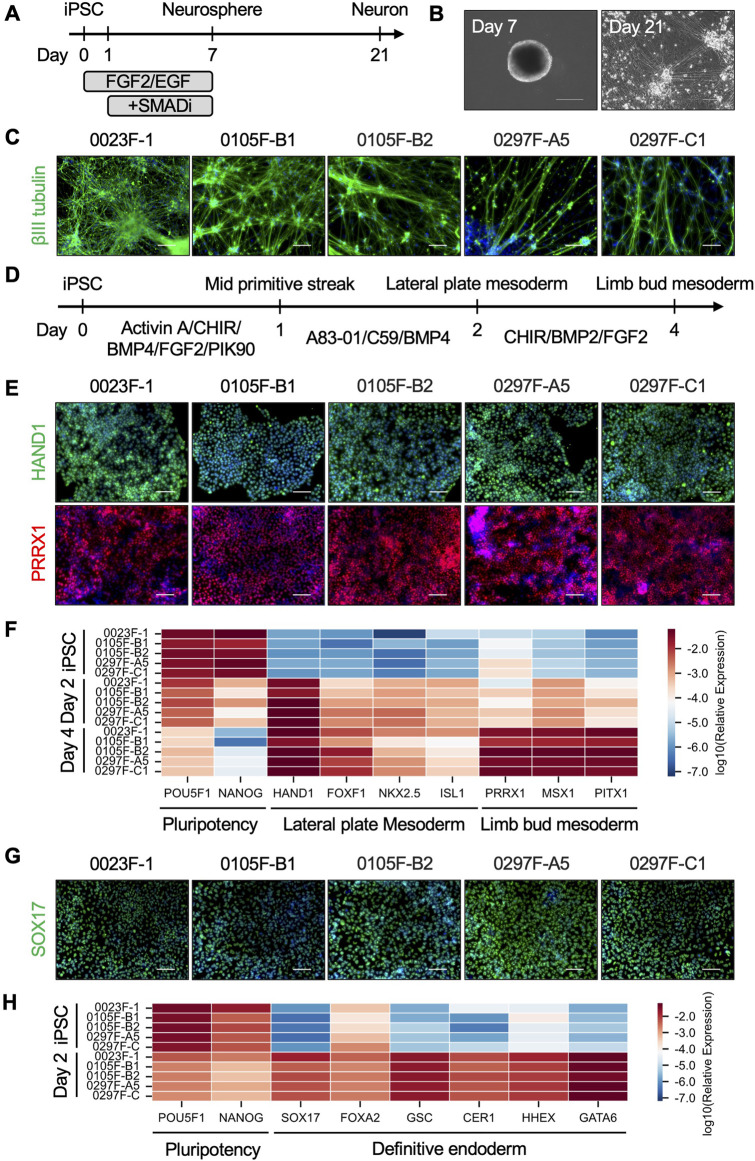
Directed differentiation of small ape iPSCs. **(A)** Schematic overview of direct neurosphere formation and the subsequent neuronal differentiation cultures. **(B)** Phase-contrast images of representative neurospheres (day 7) and neurons (day 21) derived from small ape iPSCs. **(C)** Immunofluorescence images of βIII tubulin in day 21 neurons. Nuclei were counterstained with DAPI. Scale bar: 100 μm. **(D)** Schematic design of limb bud mesoderm differentiation culture. **(E)** Immunofluorescence images of HAND1 in day 2 lateral plate mesoderm cells and PRRX1 in day 4 limb bud mesoderm cells. Nuclei were counterstained with DAPI. Scale bar: 100 μm. **(F)** Heatmap showing the expression of genes associated with pluripotency (*POU5F1*, *NANOG*), lateral plate mesoderm (*HAND1*, *FOXF1*, *NKX2.5*, *ISL1*), and limb bud mesoderm (*PRRX1*, *MSX1*, *PITX1*) in iPSCs, day 2 lateral plate mesoderm cells, and day 4 limb bud mesoderm cells determined by quantitative RT-PCR analysis. *β-ACTIN* was used as an internal control. **(G)** Immunofluorescence images of SOX17 in day 2 definitive endoderm cells. Nuclei were counterstained with DAPI. Scale bar: 100 μm. **(H)** Heatmap showing the expression of genes associated with pluripotency (*POU5F1*, *NANOG*) and definitive endoderm (*SOX17*, *FOXA2*, *GSC*, *CER1*, *HHEX*, *GATA6*) in iPSCs and day 2 definitive endoderm cells determined by quantitative RT-PCR analysis.

To investigate the differentiation potential of the small ape iPSCs, we performed directed differentiation to induce lineage-specific differentiation into the three germ layers. First, to evaluate ectodermal differentiation, we employed a direct neurosphere formation culture, which recapitulates early neural development in human and non-human primate iPSCs (Nakai et al., 2018; Kitajima et al., 2020; Nakai et al., 2022) (Figure 4A). Small ape iPSCs formed neurospheres following a 7-day suspension culture, subsequently producing βIII tubulin^+^ neurons after an additional 14 days in adherent culture conditions (Figures 4B,C). For mesodermal and endodermal differentiation, we employed stepwise protocols to induce limb bud mesoderm or definitive endoderm differentiation from iPSCs. Referring to previous reports (Loh et al., 2014; Loh et al., 2016; Ang et al., 2018; Yamada et al., 2021; Smith et al., 2023), we optimized the protocol for limb bud mesoderm differentiation, guiding sequential differentiation into the mid primitive streak (day 1), lateral plate mesoderm (day 2), and limb bud mesoderm (day 4) (Figure 4D). Immunofluorescence analyses demonstrated the efficient differentiation of HAND1^+^ lateral plate mesoderm cells on day 2 and PRRX1^+^ limb bud mesoderm cells on day 4 (Figure 4E). Quantitative RT-PCR further confirmed these findings, showing the downregulation of pluripotency markers (*POU5F1*, *NANOG*) and stepwise upregulation of lateral plate mesoderm markers (*HAND1*, *FOXF1*, *NKX2.5*, *ISL1*) and limb bud mesoderm markers (*PRRX1*, *MSX1*, *PITX1*) over the course of differentiation (Figure 4F). In a separate differentiation protocol, small ape iPSCs were directed towards definitive endoderm cells. Efficient differentiation into definitive endoderm cells was demonstrated by the expression of SOX17 protein in immunofluorescence analyses (Figure 4G) and by quantitative RT-PCR detection of definitive endoderm-associated genes (*SOX17*, *FOXA2*, *GSC*, *CER1*, *HHEX*, *GATA6*) (Figure 4H). These results demonstrate that small ape iPSCs possess robust differentiation potential, capable of generating derivatives of all three germ layers.

### 3.5 Cross-species comparison of transcriptomes among primate iPSCs

**FIGURE 5:**
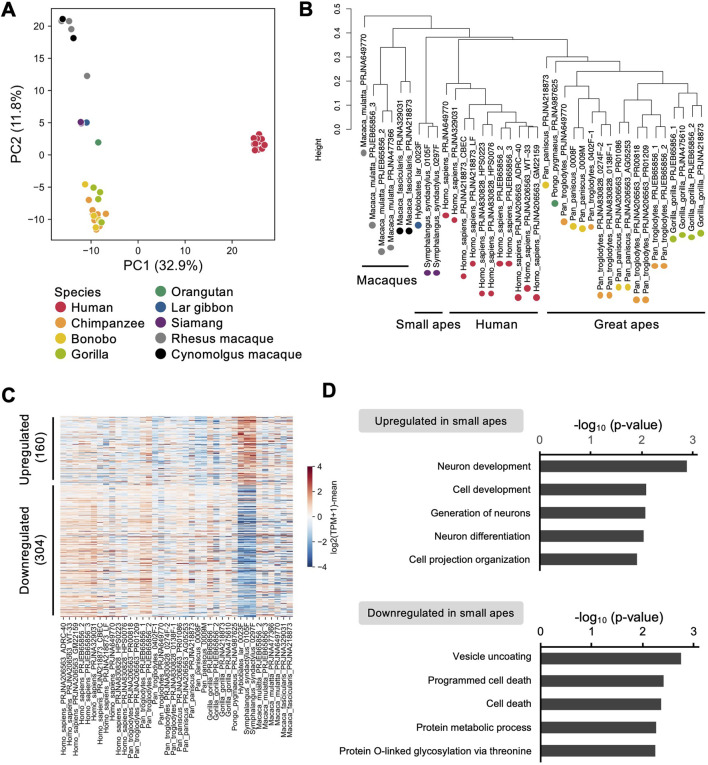
Comparative transcriptome analyses of iPSCs among primate species. **(A–D)** RNA-seq analyses of iPSCs in humans, great apes (chimpanzees, bonobos, gorillas, orangutans), small apes (lar gibbons, siamangs), and macaques (rhesus macaques, cynomolgus macaques) based on data from the current and previous studies. **(A)** PCA of the transcriptomes of iPSCs from each species. **(B)** Hierarchical clustering of the transcriptomes of iPSCs from each species. **(C)** Heatmap of genes differentially expressed between small ape iPSCs and those from other primate species. **(D)** Gene ontology analysis of genes upregulated and downregulated in small ape iPSCs compared with the levels in iPSCs from other primate species.

Finally, to address the species-specific expression signatures, we compared the gene expression profiles of small ape iPSCs with those of other primate iPSCs reported in previous studies. We constructed a dataset comprising 90 sets of sequencing data from 42 individuals across nine primate species, namely, humans (*Homo sapiens*) (Marchetto et al., 2013; Wunderlich et al., 2014; Thoma et al., 2016; Hirata et al., 2022; Roodgar et al., 2022; Ruiz-Orera et al., 2024), great apes [chimpanzees (*Pan troglodytes*), bonobos (*Pan paniscus*), gorillas (*Gorilla*), and orangutans (*Pongo pygmaeus*)] (Marchetto et al., 2013; Wunderlich et al., 2014; Hirata et al., 2022; Roodgar et al., 2022; Li et al., 2024; Ruiz-Orera et al., 2024), small apes [lar gibbons (*H. lar*) and siamangs (*S. syndactylus*)], and macaques [rhesus macaques (*Macaca mulatta*) and cynomolgus macaques (*Macaca fascicularis*)] (Wunderlich et al., 2014; Thoma et al., 2016; Roodgar et al., 2022; Ruiz-Orera et al., 2024) (Supplementary Table S4), from eight independent studies. To minimize the confounding factors unrelated to interspecies differences, hierarchical clustering was performed to identify and exclude outlier samples that clustered separately from the majority (height >0.8) (Supplementary Figure S3). Batch effects were mitigated by identifying and removing genes exhibiting study-specific expression biases (FDR <0.05) (Supplementary Table S5). This preprocessing yielded a gene expression matrix comprising 38 individuals and 5,310 genes (Supplementary Table S6). PCA of this matrix demonstrated a clear separation between human and non-human primate iPSCs along PC1. Non-human primate iPSCs were distributed along PC2 in a pattern reflecting their phylogenetic relationships (Figure 5A). Hierarchical clustering further segregated the samples into distinct clusters corresponding to humans, great apes, small apes, and macaques (Figure 5B), confirming the utility of this matrix for exploring the species-specific gene expression patterns. Differential expression analysis identified 160 upregulated and 304 downregulated genes specific to the small apes (Figure 5C; Supplementary Table S7, S8). Notably, the downregulated genes were significantly enriched for functions related to genome stability and cell death (e.g., *BUB1*, *DRAM1*, *RAD21*) (Pati et al., 2002; Jeganathan et al., 2007; Guan et al., 2015) (Figure 5D; Supplementary Tables S9, S10). These findings raise the possibility of the presence of small ape-specific regulatory mechanisms and responses to DNA damage and chromosomal rearrangements that are distinct from those observed in other primate species.

## 4 Discussion

iPSCs had been successfully established in the great apes by the early 2010s: chimpanzees (Marchetto et al., 2013; Gallego Romero et al., 2015; Kitajima et al., 2020; Lin et al., 2020; Imamura et al., 2024), bonobos (Marchetto et al., 2013; Wunderlich et al., 2014), gorillas (Wunderlich et al., 2014; Geuder et al., 2021), and orangutans (Ramaswamy et al., 2015; Geuder et al., 2021; Li et al., 2024). By contrast, reprogramming of small ape cells has proven significantly more challenging, requiring greater effort and extended timeframes (Anwised et al., 2023). During the preparation of this manuscript, another group reported the successful generation of small ape iPSCs using plasmid and SeV vectors (Bao et al., 2024). However, the small ape iPSCs have not yet been comprehensively characterized, leaving their molecular properties and potential applications insufficiently understood. In this study, we successfully reprogrammed skin fibroblasts from three small ape species using SRVs, and established five iPSC lines from one lar gibbon and two siamangs. Prior to this study, we also attempted to reprogram small ape cells using plasmid or SeV vectors. While plasmid- and SeV-based gene transduction was effective in the reprogramming of great ape and macaque cells in our previous studies (Nakai et al., 2018; Kitajima et al., 2020; Imamura et al., 2024), these attempts were not successful for small ape cells (data not shown), contrary to the findings of others (Bao et al., 2024). Both vectors allow transient transgene expression without genomic integration and are spontaneously eliminated from transduced cells. By contrast, SRVs provide robust, stable, and sustained transgene expression in the cytoplasm without triggering innate immune responses, thereby improving reprogramming efficiency (Nakanishi and Iijima, 2024). This advantage of SRVs likely contributed to our success in reprogramming the small ape fibroblasts.

Despite advancements in gene transduction, the reprogramming efficiency of small ape fibroblasts was markedly lower than that observed in humans. Most SRV-transduced cells disappeared during reprogramming, and only a few successfully reprogrammed colonies emerged (Figure 1D). This observation could be interpreted in line with previous findings that the mechanisms of DNA damage responses and cell cycle checkpoints function as the major barriers to reprogramming by triggering reprogramming-induced senescence and apoptosis of transduced cells (Lin et al., 2014). For instance, p53 and related cyclin-dependent kinase inhibitors restrict reprogramming, whereas deficiencies in these pathways enhance the efficiency of iPSC generation (Banito et al., 2009; Hong et al., 2009; Marion et al., 2009). Conversely, deficiency of *ataxia-telangiectasia mutated* (*ATM*) or *53BP1*−both critical for DNA damage responses and repair−impairs reprogramming efficiency and leads to the accumulation of chromosomal abnormalities during iPSC generation (Marion et al., 2009; Kinoshita et al., 2011). Small apes have undergone exceptionally rapid and large-scale chromosomal rearrangements (Carbone et al., 2006; Capozzi et al., 2012), which are partially attributed to the insertion of the small ape-specific retrotransposon *LAVA*. The small ape genome contains 600–1,200 *LAVA* insertions, approximately half of which are located within or near genes regulating the cell cycle, DNA repair, and chromosome segregation (Carbone et al., 2014; Okhovat et al., 2020). These genomic alterations might exacerbate reprogramming-induced senescence and apoptosis, thereby impairing cellular reprogramming in small ape cells. Supporting this hypothesis, enrichment of cell death-related genes was observed among the genes expressed at lower levels in small apes than in other primates (Figure 5D). These genes included *RAD21*, a key regulator of chromosome stability and cell death (Pati et al., 2002), and *BUB1*, a mediator of cell death induced by chromosome missegregation (Jeganathan et al., 2007). Notably, a *LAVA* insertion was identified near the *BUB1* gene locus (Okhovat et al., 2020). Furthermore, considering that not all SRV^+^ reprogrammed cell lines yielded SRV-free iPSCs but instead underwent differentiation upon siRNA treatment (data not shown), further optimization is required to overcome reprogramming barriers and reliably establish iPSCs in small apes.

The transcriptome profiles of small ape iPSCs revealed that reprogramming of small ape fibroblasts led to the upregulation of pluripotency-associated genes and TEs, consistent with observations in other primate iPSCs (Marchetto et al., 2013; Grow et al., 2015; Kunitomi et al., 2022). Notably, upregulated TEs included *LTR7* and *HERVH* (Figure 3C), which are highly expressed in human iPSCs and play a critical roles in reprogramming and differentiation (Santoni et al., 2012; Lu et al., 2014; Ohnuki et al., 2014; Zhang et al., 2019; Han et al., 2023). These findings confirm the successful reprogramming of small ape cells and highlight conserved pluripotency characteristics across primate iPSCs. In addition, we observed species-specific expression of genes and TEs, including the small ape-specific *LAVA* (Figures 3D, 5C). Because *LAVA* can function as an enhancer element (Okhovat et al., 2020), its insertion may alter the expression of nearby genes in specific cell types of small apes. The *LAVA* expression detected in this study suggests an active chromatin state at these insertion sites (Figure 3D). As noted above, *LAVA*-induced alterations in genes associated with cell death and genomic stability may have facilitated the chromosomal rearrangements unique to small ape evolution. Therefore, small ape iPSCs offer valuable opportunities to investigate the regulation and functional role of TEs, particularly *LAVA*, in genome evolution.

Directed differentiation cultures of iPSCs have enabled detailed analyses of the molecular and cellular foundation underlying the phenotypic evolution of humans and great apes (Anwised et al., 2023; Pollen et al., 2023). For example, iPSC-based neural differentiation cultures have revealed interspecies heterochronic differences such as variation in the progression of maturation and migration (Marchetto et al., 2019; Benito-Kwiecinski et al., 2021). We have also developed the direct neurosphere formation culture to induce early neural development from human, chimpanzee, and macaque iPSCs, uncovering delayed neuronal differentiation along the evolutionary trajectory toward humans (Nakai et al., 2018; Kitajima et al., 2020; Nakai et al., 2022). Here, we demonstrated that direct neurosphere formation culture is applicable to small ape iPSCs, bridging the phylogenetic gap between great apes and catarrhine monkeys. This advancement provides critical insights into the evolutionary properties of neural development from monkeys to hominoids for future studies.

It is also notable that we successfully induced limb bud mesoderm from small ape iPSCs. Small apes have extremely long forelimbs and well-developed shoulder muscles as an adaptive evolution to brachiation (Hollihn, 1984; Michilsens et al., 2009). Mechanisms underlying their unique forelimb development have primarily been explored through whole-genome analyses, which have identified genomic regions associated with these phenotypes. For example, positive selection was identified in small apes for an amino acid substitution in the TBX5 protein (Carbone et al., 2014), which is expressed in the future forelimb position of lateral plate mesoderm to initiate limb bud growth (Agarwal et al., 2003; Rallis et al., 2003; Hasson et al., 2007). Moreover, small ape-specific accelerated regions, which feature rapid DNA substitutions driving accelerated genome evolution, are enriched near genes associated with the development of growth plate cartilages. Some of these accelerated regions also regulate the expression of limb development-related genes such as *DLX5* and *EMX2* (Bi et al., 2023). These findings suggest that coding and regulatory genome alterations specific to small apes have played a role in their forelimb development. On the other hand, extant apes shared anatomical traits associated with suspensory locomotion, such as relatively longer forelimbs, which are thought to have evolved independently within each lineage (Larson, 1998). The limb bud mesoderm-directed differentiation culture of their iPSCs provides an essential platform for investigating the molecular foundations underlying limb development and evolution in hominoids (Young et al., 2010; Cotney et al., 2013; Tsutsumi and Eiraku, 2023), as exemplified by an iPSC-based study of interspecies differences in craniofacial morphology (Prescott et al., 2015).

## Data Availability

The datasets presented in this study can be found in online repositories. RNA-seq data of small ape cells established in this study can be found in GEO with accession number GSE282905.
