# Supplementary figures and images for: Generation and characterization of induced pluripotent stem cells of small apes
Source: Front Cell Dev Biol. 2025 Mar 19;13:1536947. doi: 10.3389/fcell.2025.1536947 (PMC11961953; doi:10.3389/fcell.2025.1536947)

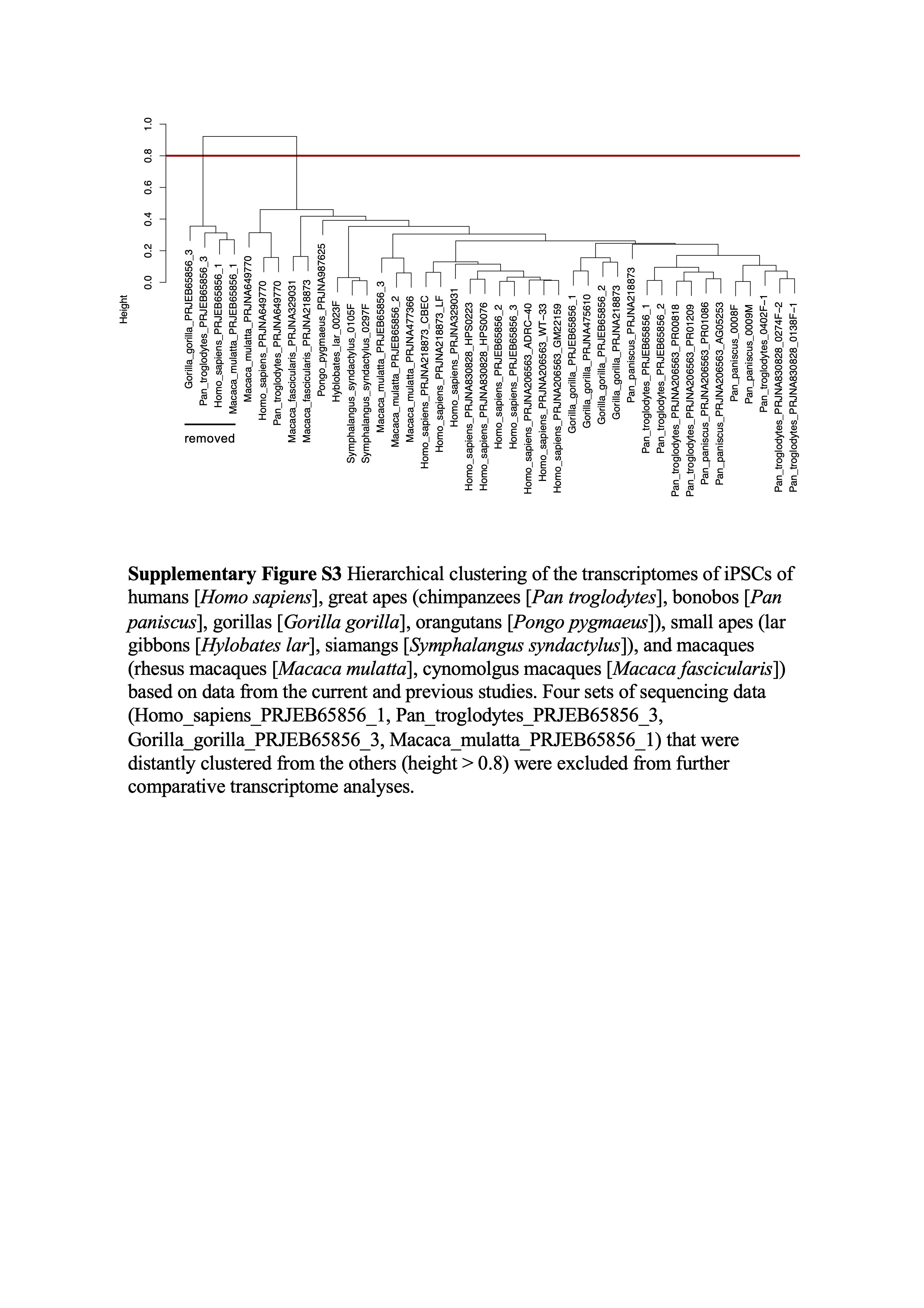

Supplement: Supplementary file 1 [file Image3.jpeg]

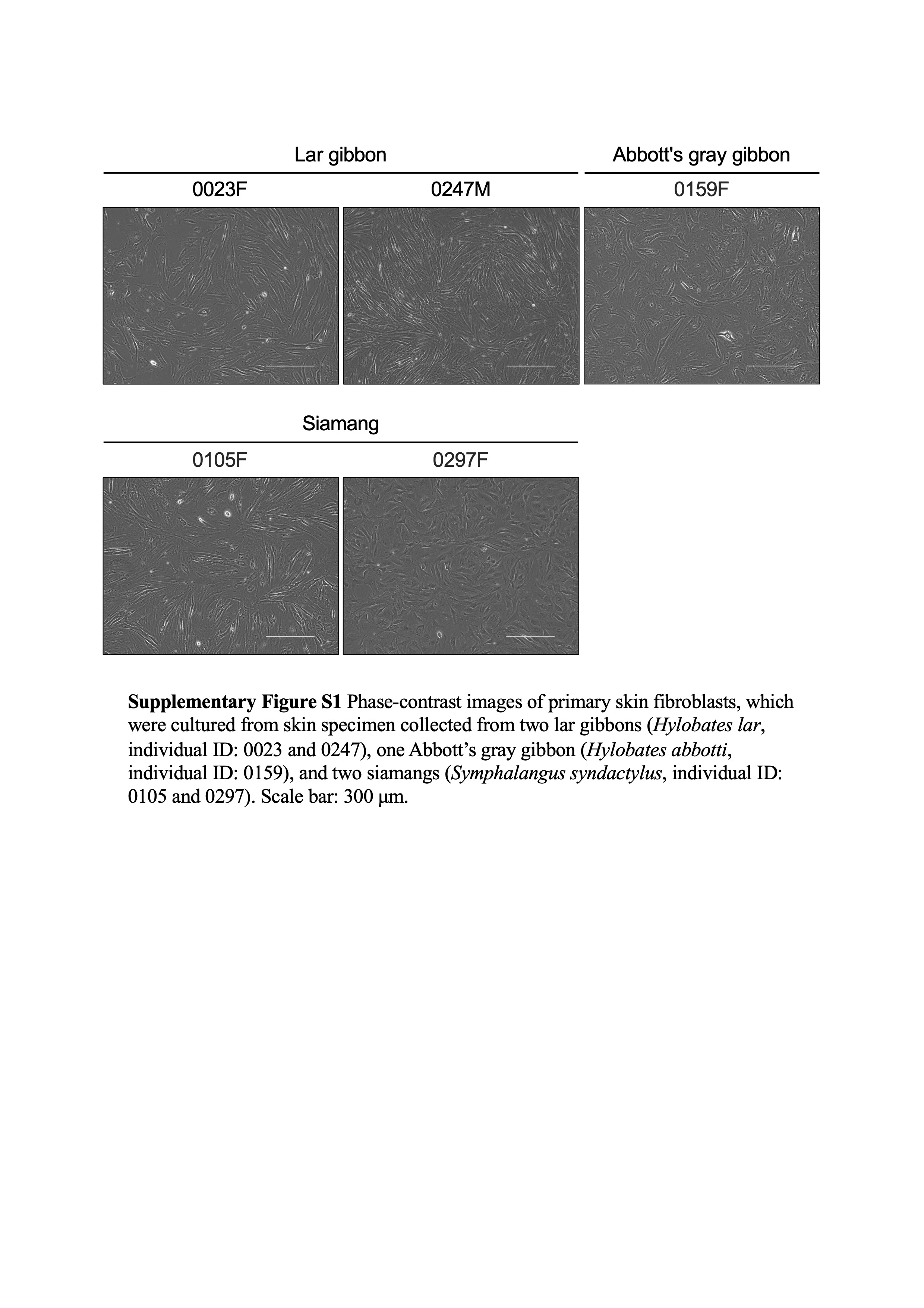

Supplement: Supplementary file 2 [file Image1.jpeg]

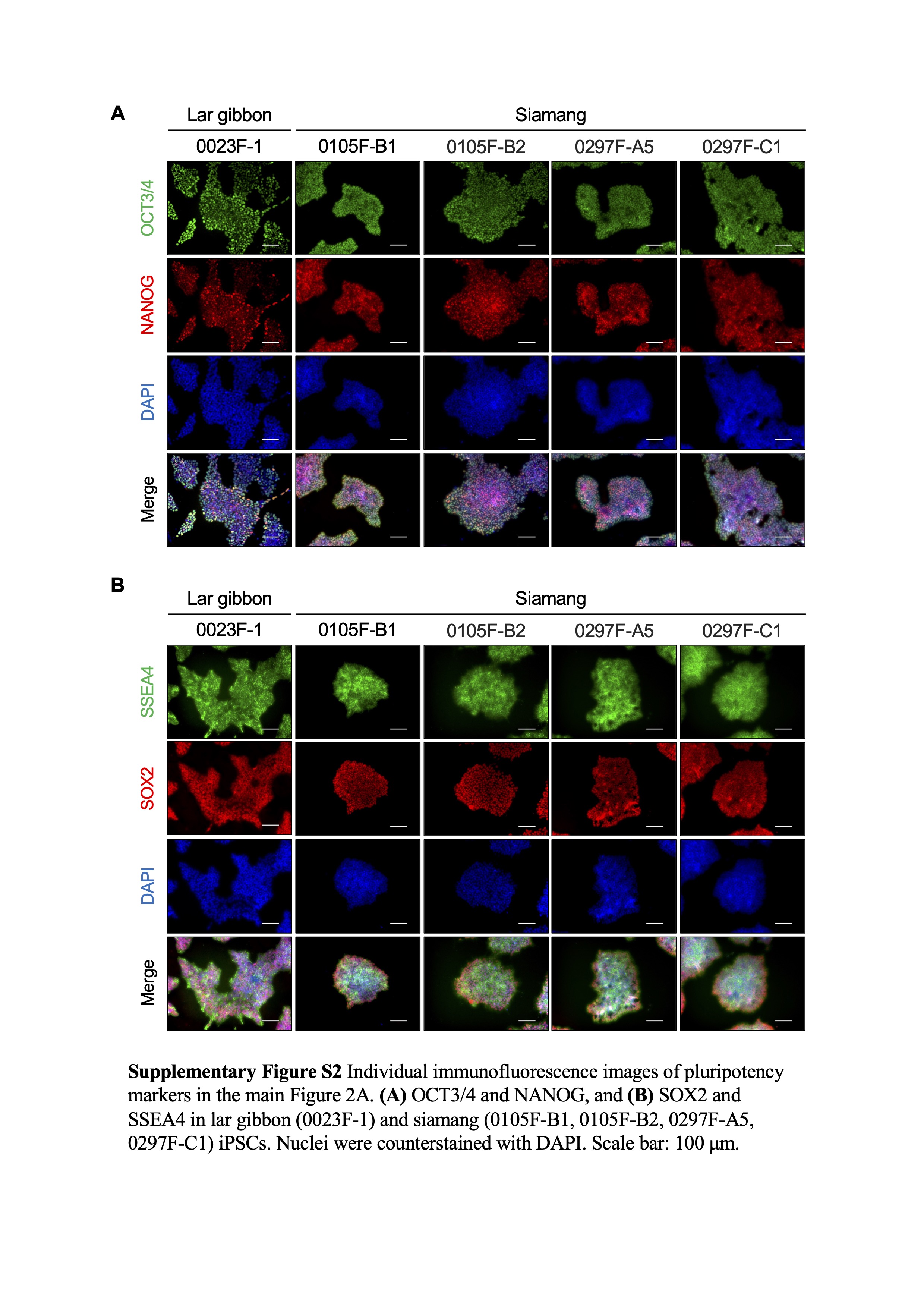

Supplement: Supplementary file 3 [file Image2.jpeg]
